# Iron Status and NAFLD among European Populations: A Bidirectional Two-Sample Mendelian Randomization Study

**DOI:** 10.3390/nu14245237

**Published:** 2022-12-08

**Authors:** Cenqin Liu, Yishu Chen, Zhixin Zhang, Jiarong Xie, Chaohui Yu, Lei Xu, Youming Li

**Affiliations:** 1 Department of Gastroenterology, Ningbo Hospital, Zhejiang University, Ningbo 315010, China; 2 Department of Gastroenterology, The First Affiliated Hospital, College of Medicine, Zhejiang University, Hangzhou 310003, China; 3 Department of Gastroenterology, Ningbo First Hospital, Ningbo 315010, China; 4 School of Medicine, Ningbo University, Ningbo 315010, China

**Keywords:** mendelian randomization, NAFLD, serum ferritin, iron, TSAT, TIBC

## Abstract

**Background and aim:** Previous observational studies have suggested a paradoxical relationship between iron status and the risk of non-alcoholic fatty liver disease (NAFLD). Observed associations in these epidemiological studies fail to show sequential temporality and suffer from problems of confounding. Therefore, we performed a bidirectional two-sample Mendelian randomization (MR) to evaluate the relationship between serum iron status and NAFLD. **Methods:** The inverse weighted method (IVW) meta-analysis with the fixed-effect model was the main method to estimate the relationship between iron status, including serum ferritin, iron, transferrin saturation (TSAT) and total iron-binding capacity (TIBC), and NAFLD. Weighted median, penalized weighted median, and MR Robust Adjusted Profile Score (MR RAPS) methods were used as additional analyses. Sensitivity analyses were performed with Cochran’s Q test, MR–Egger regression, Steiger filtering, and the MR PRESSO test. **Results:** Iron status, including serum ferritin, iron, and TSAT, was associated with an increased risk of NAFLD (odds ratio (OR) (95% confidence interval (CI)): 1.25 (1.06, 1.48); 1.24 (1.05, 1.46), 1.16 (1.02, 1.31), respectively). In contrast, minimal effects of NAFLD on serum ferritin, iron, TSAT, and TIBC were observed (OR (95% CI): 1.01 (1.00, 1.02), 1.01 (1.00, 1.02), 1.03 (1.01, 1.05), 1.03 (1.01, 1.05), respectively). **Conclusions:** Our findings corroborated the causal associations between serum ferritin, iron, TSAT, and NAFLD, which might suggest the potential benefits of iron-related therapy. In addition, NAFLD might, in turn, slightly affect iron homeostasis indicated as serum ferritin, iron, TSAT, and TIBC, but this needs to be further confirmed.

## 1. Introduction

Non-alcoholic fatty liver disease (NAFLD) is the most common chronic liver disorder affecting one-quarter of individuals worldwide and is a leading cause of cirrhosis and hepatocellular carcinoma [1]. The predominant drivers of NAFLD vary substantially, as the liver disease component of metabolic syndrome has been increasingly observed to be associated with iron metabolism [1,2,3]. Iron is an essential trace element involved in complex physiological processes, including oxygen transport, cellular respiration, and DNA synthesis, which on the one hand, protects the host from infection, but on the other hand, stimulates oxidative damage [4,5]. 

Iron status distribution disorders have been reported in many studies as a contributor to the development of NAFLD [6,7,8]. Several studies showed that hepatocellular iron deposition and higher serum ferritin were associated with an increased risk of hepatic fibrosis [9,10]. However, iron deposition was not observed to be associated with the severity of liver damage in another study that involved 66 patients with biopsy-proven NAFLD [11]. Other studies showed a prevalent iron deficiency in NAFLD [12] and suggested that a higher level of serum iron was negatively associated with the risk of NAFLD [13]. A randomized controlled trial also showed that reduction in serum ferritin did not improve liver enzymes or hepatic lipid accumulation in patients with NAFLD [14]. This topic seems to be controversial.

Observational studies might be affected by their multiple contributing factors because of the slow progression and the heterogeneity of NAFLD or reverse causation bias [1]. Mendelian randomization (MR) studies use genetic variants, such as single nucleotide polymorphisms (SNPs), which are naturally, randomly distributed during meiosis at conception, and, therefore, could reduce confounders and reverse causation bias in epidemiological studies to estimate their possible relationship between exposures and outcomes [15]. In the present work, we aimed to perform a bidirectional two-sample MR study to assess the possible associations between iron status, including ferritin, serum iron, transferrin saturation (TSAT), total iron-binding capacity (TIBC), and the risk of NAFLD.

## 2. Materials and Methods

**Figure 1:**
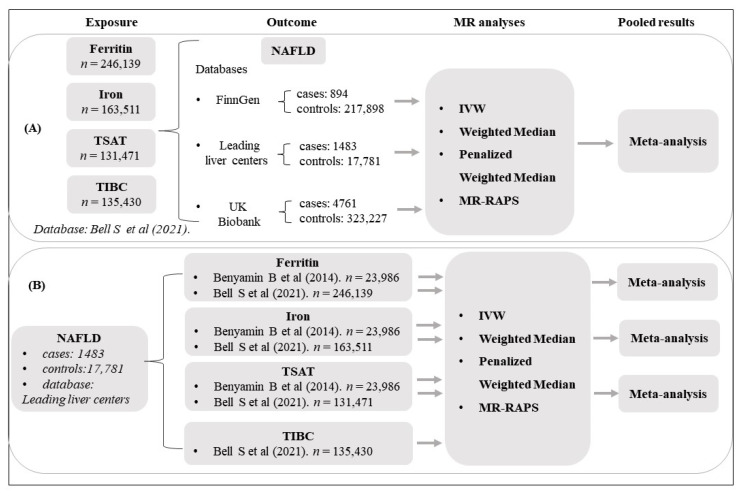
Schematic overview of the study design. (**A**) MR study from iron status, including serum ferritin, iron, transferrin saturation (TSAT), and total iron-binding capacity (TIBC) to non-alcoholic fatty liver disease (NAFLD): SNPs for iron status were selected from the Bell Steven et al. database as exposure variables, whereas summary statistics of gene-NAFLD associations were retrieved separately from three databases: FinnGen, several leading European tertiary liver centers, and UK Biobank. MR analyses were conducted per outcome database and were subsequently meta-analyzed to generate pooled estimates. (**B**) MR study from NAFLD to iron status: SNPs for NAFLD were selected from a GWAS study based on several leading European tertiary liver centers as exposure variables, whereas summary statistics of gene-iron-status associations were retrieved separately from two databases (except for TIBC): Benyamin Beben et al. [16] and Bell Steven et al. [17], MR analyses were performed per outcome database and were subsequently meta-analyzed to generate pooled estimates.

We performed a bidirectional two-sample MR to investigate the potential bidirectional causal relationship between iron status and NAFLD. For summary statistics of the outcome, we tried to search for as many genome-wide association studies (GWAS) on iron status and NAFLD from different European populations as possible to avoid an underpowered sample size. Figure 1 shows an overview of the study design. All data were obtained from published studies and publicly available databases. Therefore, no additional ethics approval or consent to participate was required.

### 2.1. Defining Genetic Instruments

The genetic variants associated with exposures of interest, including serum ferritin, iron, TSAT, and TIBC, were obtained from a public GWAS [17] The GWAS meta-analysis of serum ferritin (*n* = 246,139), iron (*n* = 163,511), TSAT (*n* = 131,471), and TIBC (*n* = 135,430) involved three cohorts from Iceland, the UK, and Denmark, in which effects were given in units of standard deviation. Independent genome-wide significant (*p* < 5 × 10^−8^) genetic instruments for the four iron status indicators were constructed (linkage disequilibrium (LD), r^2^ < 0.001 based on the phase 3 data of the European 1000 Genome Project reference panel).

Genetic variants associated with NAFLD were obtained from a GWAS study [18] which involved 1483 European cases and 17,781 controls. All NAFLD cases were recruited from clinics at several leading European tertiary liver centers and had undergone a liver biopsy, where biopsy specimens were assessed according to accepted criteria by experienced liver pathologists. This NAFLD case-control analysis finally confirmed 12 significant SNPs that were included in our study. The strength of the genetic instruments for exposure was quantified using F statistics, which was calculated as (beta/se)^2^, and a value of above 10 was considered sufficient [19]. The proportions of variance explained by exposures (R^2^) were calculated in R software. 

### 2.2. Genetic Associations with Outcomes

The summary statistics of gene–outcome associations for NAFLD were obtained from three databases of FinnGen, several leading European tertiary liver centers, and UK Biobank (UKB). The FinnGen database provided data of 894 cases with NAFLD that were mainly defined by the International Statistical Classification of Diseases and Related Health Problems (ICD) 10 and 217,898 controls (www.gwas.mrcieu.ac.uk, accessed on 22 August 2022). The second study, based on several leading European tertiary liver centers, involved 1483 European NAFLD cases with a median body mass index (BMI) of 35.2 kg/m^2^ and underwent a liver biopsy and 17,781 genetically matched controls, adjusted for the incorporation of the top 5 principal components as covariates [18]. In the UKB GWAS study, NAFLD was defined by the ICD and Clinical terminology system used in UK Primary Care settings, including 4761 cases and 373,227 controls (mean BMI was 31.4 kg/m^2^ vs. 27.4 kg/m^2^, respectively), adjusted for age, sex, and the first 20 genetic principal components [20].

Summary statistics for iron status were obtained from the two GWAS studies. Benyamin Beben et al. analyzed genetic association data on ferritin, iron, and TSAT after adjustment for age, principal component scores, and other-study specific covariates from 11 cohorts involving 23,986 subjects of European ancestry mainly came from the Australian cohort (11,694 individuals), German cohort (3443 individuals), and so on [16]. GWAS on TIBC was not available in the study of Benyamin Beben et al. Another study published in 2021 by Bell Steven et al. performed a meta-analysis of three GWAS on serum ferritin (*n* = 246,139), iron (*n* = 163,511), TSAT (*n* = 131,471), and TIBC (*n* = 135,430) from Iceland, the UK, and Denmark, adjusted for covariates of sex, age, and country of birth, as well as principal components [17]. No overlapping cohorts were observed in these two studies. 

### 2.3. MR Estimation

Palindromic SNPs were discarded for further MR analyses in data of exposure and outcome harmonization [21], which were performed separately in each outcome database, and the individual estimates were subsequently combined using a fixed-effect meta-analysis. Inverse variance weighted (IVW) meta-analysis as the primary analysis was used to measure the association between iron status and NAFLD. The weighted median method, penalized weighted median method, and MR Robust Adjusted Profile Score (MR RAPS) method as additional analyses with different assumptions were conducted, which could provide effect estimates when 50% of the weight came from valid IVs and reduce the influence of variants with heterogeneity and pleiotropy [22,23], respectively. Cochran’s Q statistic was calculated to quantify heterogeneity, and potential directional pleiotropy could be estimated by intercept of the MR-Egger regression and MR pleiotropy residual sum and outlier (MR-PRESSO). In addition, MR Steiger filtering was performed to assess whether the instrumental variables (IVs) associated with exposure directly affected the outcome, which means a “FALSE” direction and implies possible reverse causation [24]. Therefore, IVs that were identified as outliers by the MR-PRESSO estimator or with a “FALSE” direction were removed, and the MR causal estimation was reassessed. All analyses were performed using R software (version 4.1.2) with the “TwoSampleMR”, “MRPRESSO”, and “meta” packages.

## 3. Results

### 3.1. Iron Status to NAFLD

The number of independent SNPs associated with iron status ranged from 8 to 27, explaining 1.19% to 2.72% of the variance, and each SNP was greater than the empirical threshold 10 of the F statistics (Table 1).

**Figure 2:**
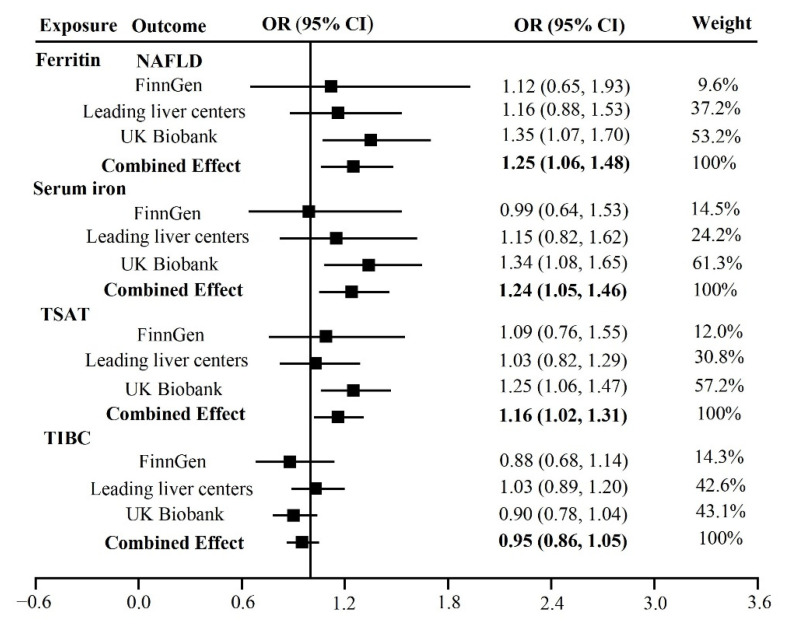
Causal associations between iron status and NAFLD risk.

IVW meta-analyses showed that the pooled odds ratios (ORs) for NAFLD of genetically predicted per log-OR increase in serum ferritin, iron, TSAT, and TIBC were 1.25 (95% confidence interval (CI): 1.06, 1.48), 1.24 (95% CI: 1.05, 1.46), 1.16 (95% CI: 1.02, 1.31), 0.95 (95% CI: 0.86, 1.05), respectively (Figure 2). Weighted median, penalized weighted median, and MR RAPS as the supplementary estimators also revealed similar statistical associations between ferritin, TIBC, and NAFLD (Table S1). 

In the sensitivity analyses, the results of serum iron on NAFLD of the UKB database obtained from the weighted median and penalized estimators were different from those from IVW and MR RAPS. This might be because the weighted median method is based on the hypothesis that more than half of the SNPs are valid, while the IVW method is not. No pleiotropic effect was observed in the final analysis by MR-Egger intercept and MR PRESSO global test after we removed the outliers, and all IVs included were with a “TRUE” direction in the Steiger filtering. Only in the analysis of TIBC on NAFLD, which came from the UKB database, was heterogeneity of each instrument estimation detected as evaluated by Cochran’s Q test statistics. However, the recommended median-based estimation in this circumstance gave a similar result to the IVW method, according to a previous study [25].

### 3.2. NAFLD to Iron Status

Twelve SNPs as genetic instruments for NAFLD explained 3.42% of the exposure variance, with the median F statistics of 41.9.

**Figure 3:**
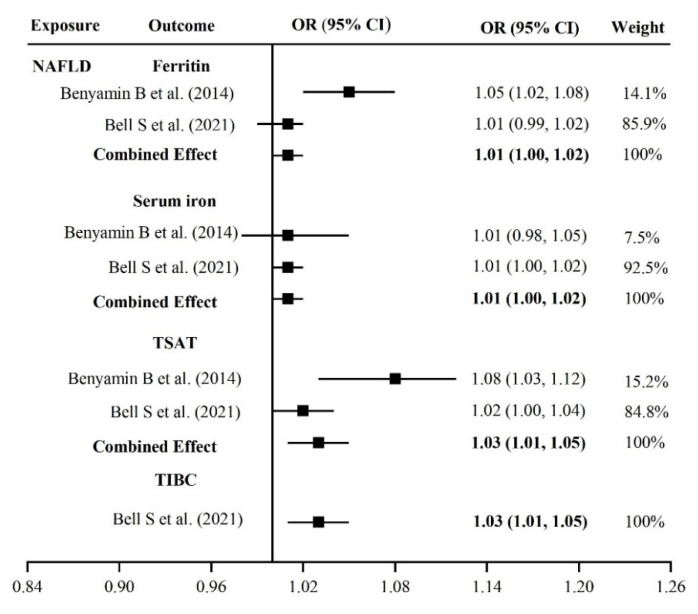
Causal associations between NAFLD and iron status [17,16].

Overall, our results of IVW meta-analyses showed minimal effects of NAFLD on iron status, with combined ORs for ferritin, iron, and TSAT of 1.01 (95% CI: 1.00, 1.02), 1.01 (95% CI: 1.00, 1.02), 1.03 (95% CI: 1.01, 1.05), respectively (Figure 3). In the analysis of TIBC, which was obtained from the Bell Steven et al. database, a similarly minimal effect was observed by the IVW estimator (OR: 1.03, 95% CI: 1.01, 1.05).

Weighted median, penalized weighted median, and MR RAPS estimators provided results similar to those using the IVW method in each database (Table S1). There were outliers in the analyses of NAFLD on ferritin, TSAT, and TIBC when we performed the MR PRESSO test. Therefore, MR estimates were reassessed, and the final results were based on the absence of pleiotropy and heterogeneity. In addition, no other outliers with the “FLASE” direction were detected by Steiger filtering.

## 4. Discussion

Our study showed that the iron status, including serum ferritin, iron, and TSAT, was associated with an increased risk of NAFLD. In addition, NAFLD may also affect iron homeostasis, resulting in elevated ferritin, iron, TSAT, and TIBC. 

Previous studies on the associations between iron status and NAFLD so far have been controversial. As far as we know, this study is the first to utilize the MR method based on several GWAS analyses with a larger sample size, suggesting a possible causal association between serum ferritin, iron, TSAT, and NAFLD. The liver serves as the major organ for iron metabolism and is also affected by iron homeostasis [26]. According to a study based on the post-mortem autopsies of 187 patients, chronic intravenous administration of iron, even with the minimum recommended dosage, increased iron deposition in the liver [27]. It is worth noting that patients with hepatocellular iron deposition were almost universally considered to be associated with an increased risk of hepatic fibrosis [7,10]. Gao’s recent study published in 2022 demonstrated that iron overload in hepatic stellate cells resulted in the overproduction of reactive oxygen species that promote fibrogenic activation among patients with NAFLD [6]. An earlier study performed in mice also suggested that increased hepatic iron facilitated the significant upregulation of the transcripts of seven enzymes in the cholesterol biosynthesis pathway, by which iron could contribute to the development of fatty liver disease or lipotoxicity [28]. In addition, NAFLD has been recognized to have a close bidirectional association with metabolic syndrome, particularly obesity and type 2 diabetes [1]. In an experiment with mice, iron was identified to aggravate the related symptoms of type 2 diabetes and insulin resistance which was a key operative mechanism involved in the development of NAFLD [29,30].

Serum ferritin, as a marker of liver iron stores, was identified as an independent predictor of NAFLD as early as 2012 [9], and hyperferritinemia was often even the first abnormality leading to medical attention in such patients [31]. The most frequent inherited form of iron overload, hereditary hemochromatosis, is caused by mutations of the hemochromatosis gene (HFE) and indicated by increased serum ferritin and elevated TSAT, which is the earliest biochemical sign observed in all hemochromatosis subtypes when its level is >45% [32]. The HFE SNP, rs1800562 (C282Y) included in this study is known to cause a serious type 1 hereditary hemochromatosis, which most commonly affects the liver and significantly increases the risk of developing cirrhosis when serum ferritin exceeds 1000 ng/mL at diagnosis, according to The American College of Gastroenterology (ACG) Clinical Guideline [33]. Additionally, dysmetabolic iron overload syndrome, as one of the acquired forms of iron overload, is characterized by increased ferritin concentrations and fatty liver, where insulin resistance remains the most likely mechanism involved in the progression of both diseases [34]. A mice-related study conducted by Mayneris–Perxachs et al. suggested another possible reason that serum ferritin levels positively influenced liver fat accumulation through the gut microbiome [8]. However, although our study observed a protective effect of TIBC on NAFLD, it was not statistically significant enough to imply a possible causal relationship. TIBC indicated the maximum amount of iron necessary to saturate all available transferrin iron-binding sites; therefore, it correlated well with transferrin concentration [8]. Yu et al. suggested that patients with liver cirrhosis had significantly lower serum transferrin and higher hepatic iron and lipid peroxidation, indicating the benefit of transferrin in maintaining liver function [26]. Together, these results might support the plausibility of our findings.

NAFLD might lead to increased levels of serum ferritin, iron, TSAT, and TIBC, although these effects were obviously weaker than those of iron status (serum ferritin, iron, and TSAT) on NAFLD. For possible reasons, a single-nucleotide polymorphism, rs738409 (causing an isoleucine-to-methionine substitution at position 148, I148M) in the *patatin-like phospholipase domain-containing protein 3 (PNPLA3)* gene is strongly associated with steatosis in patients with NAFLD [35]. However, rs738409 *(PNPLA3),* as the most significant IV associated with NAFLD in our study, was discarded for further MR analyses because it was identified as the palindromic SNP in the analysis of harmonizing exposure and outcome data [18,21]. Additionally, in a study involving 849 liver biopsy samples, subjects with stainable hepatic iron had higher ferritin, serum iron, TSAT, and TIBC [7], which may be consistent with what we observed in our study. However, the causal effects were minimal, and the lower limit of the 95% confidence interval was close to 1. For these possible reasons, markers of systemic iron hemostasis were no longer coupled to the iron status of key metabolic organs, such as the liver, during metabolic dysfunction [36], which might mean that an increase in liver iron does not necessarily lead to a similarly increased serum iron. A previous study observed a frequent iron deposition in the liver but normal peripheral iron parameters in individuals with NAFLD, which confirmed this possibility [11]. In addition, serum ferritin was determined mainly by hepatic iron but differed according to fibrosis stage, increasing in early to moderate disease and declining in cirrhosis [37]. Although we searched two databases for iron status, the sample size was still insufficient compared with the outcome data of NAFLD, and in the meta-analysis of NAFLD on ferritin and TSAT, we also observed a non-negligible heterogeneity (Table S2). Anyhow, this result should be interpreted with caution and further evaluated.

This MR study with an expanded sample size minimized the risk of confounding and added strong causal evidence to previous observational and experimental studies, that is, high levels of iron status, including serum ferritin, iron, and TSAT, promote NAFLD, which might, in turn, slightly affect iron homeostasis indicated as serum ferritin, iron, TSAT, and TIBC. The effects of iron depletion have been demonstrated to significantly improve insulin resistance and have a protective effect against NAFLD and type 2 diabetes [34]. Therefore, the benefits of regular phlebotomy or iron chelation seen with hemochromatosis may potentially be extended in specific groups of patients with high physiological iron levels.

Despite the above advantages, some limitations should also be acknowledged. First, we only evaluated the effects among the European population due to the design principles of Mendelian randomization studies, which limited the generalizability to other ethnic groups. Second, although three cohorts performed strict inclusion criteria, liver biopsy was performed in only one cohort, and the diagnosis of NAFLD in the other two datasets was conducted in clinical settings. Therefore, the accuracy and the content of NAFLD may be heterogenous among them. In addition, we noticed that individual databases had a larger weight in the meta-analysis of iron status and NAFLD. For these possible reasons, when we performed the forward analysis, NAFLD as a binary outcome variable had more cases in the database of UK Biobank, in which MR estimation had a narrower confidence interval. For the reverse analysis, iron status as the outcome variable obtained from Bell S et al. [17] had a larger sample size, which was about five to ten times the size of that from Benyamin B et al. [16]. Third, we found a minimal causal effect of NAFLD on serum iron status. However, as discussed above, different fibrosis stages had different effects on iron homeostasis. NAFLD in this study, based on the available GWAS data, was a binary variable rather than a disease course, which means that we did not know whether the more detailed stage or activity score of NAFLD played a different role. Finally, although we did our best to search for available GWAS data and performed analyses on several databases except for TIBC on NAFLD, the sample size involved in this study might not be sufficient. More studies on iron status or with a larger number of NAFLD cases would be helpful for further confirmation.

## 5. Conclusions

In conclusion, our findings corroborated a causal association between iron status and NAFLD, which might draw attention to patients who have higher levels of serum ferritin, iron, and TSAT in the earliest stage of disease and highlight the potential benefits of iron-related therapeutics in NAFLD. In addition, NAFLD may act as a “cause” to affect iron homeostasis. Further investigations will be instructive. 

## Figures and Tables

**Table 1:** Summary of exposure factors.

Exposure	NSNP	F Statistic	R^2^ (%)
		Median (Min, Max)	
Ferritin	27	57.4 (30.8, 513.6)	1.19
Iron	12	52.0 (32.7, 1373.9)	2.08
TSAT	8	171.3 (42.7, 1373.9)	2.72
TIBC	13	44.5 (30.8, 1373.9)	2.41
NAFLD	12	41.9 (29.5, 219.1)	3.42

## Data Availability

All the data are available in the published article and databases.

## Supplementary Materials

The following supporting information can be downloaded at: https://www.mdpi.com/article/10.3390/nu14245237/s1, Table S1: Effect estimates of the associations between iron status and NAFLD; Table S2: Heterogeneity and test for overall effect of the IVW meta-analysis.

## Abbreviations

NAFLD	non-alcoholic fatty liver disease
TSAT	transferrin saturation
TIBC	total iron-binding capacity
MR	Mendelian randomization
SNPs	single nucleotide polymorphisms
GWAS	genome-wide association studies
IVW	inverse variance weighted
MR RAPS	MR Robust Adjusted Profile Score
MR-PRESSO	MR pleiotropy residual sum and outlier
IVs	instrumental variables
