# Supplementary material for: Iron Status and NAFLD among European Populations: A Bidirectional Two-Sample Mendelian Randomization Study
Source: Nutrients. 2022 Dec 8;14(24):5237. doi: 10.3390/nu14245237 (PMC9788387; doi:10.3390/nu14245237)
Supplement: Supplementary file 1 [file nutrients-14-05237-s001.zip › nutrients-2019229-supplementary.pdf]

Table S1 Effect estimates of the associations between iron status and NAFLD.

	No. of SNPs	IVW		Weighted Median	Penalized Weighted Median	MR RAPS	MR-Egger	MR PRESSO
		OR (95% CI)	Q test *p value*	OR (95% CI)	OR (95% CI)	OR (95% CI)	Intercept *p value*	*p* for global test
Ferritin on NAFLD								
FinnGen	26	1.12 (0.65, 1.93)	0.83	1.13 (0.53, 2.40)	1.13 (0.53, 2.37)	1.12 (0.64, 1.94)	0.62	0.16
Leading liver centers	20	1.16 (0.88, 1.53)	0.26	1.12 (0.80, 1.57)	1.12 (0.81, 1.56)	1.17 (0.90, 1.51)	0.91	0.37
UK Biobank	24	1.35 (1.07, 1.70)	0.89	1.51 (1.09, 2.09)	1.51 (1.09, 2.10)	1.35 (1.07, 1.71)	0.22	0.88
**Meta-analysis**		1.25 (1.06, 1.48)	-	1.29 (1.03, 1.61)	1.28 (1.03, 1.60)	1.25 (1.06, 1.48)	-	-
Iron on NAFLD								
FinnGen	10	0.99 (0.64, 1.53)	0.66	0.90 (0.55, 1.49)	0.90 (0.54, 1.51)	0.99 (0.63, 1.55)	0.68	0.69
Leading liver centers	9	1.15 (0.82, 1.61)	0.28	1.35 (0.88, 2.08)	1.53 (0.99, 2.36)	1.15 (0.85, 1.56)	0.23	0.30
UK Biobank	11	1.34 (1.08, 1.65)	0.15	1.17 (0.88, 1.55)	1.03 (0.79, 1.35)	1.45 (1.17, 1.79)	0.33	0.19
**Meta-analysis**		1.24 (1.05, 1.46)	-	1.15 (0.93, 1.43)	1.10 (0.90, 1.36)	1.29 (1.10, 1.52)	-	-
TSAT on NAFLD								
FinnGen	7	1.09 (0.76, 1.55)	0.77	1.05 (0.67, 1.64)	1.05 (0.70, 1.58)	1.09 (0.76, 1.56)	0.82	0.75
Leading liver centers	7	1.03 (0.82, 1.29)	0.46	0.90 (0.69, 1.18)	0.90 (0.69, 1.17)	1.03 (0.82, 1.29)	0.25	0.39
UK Biobank	8	1.25 (1.06, 1.47)	0.13	1.19 (1.01, 1.41)	1.19 (1.01, 1.40)	1.30 (1.12, 1.51)	0.42	0.27
**Meta-analysis**		1.16 (1.02, 1.31)	-	1.10 (0.96, 1.25)	1.09 (0.96, 1.25)	1.20 (1,06, 1.34)	-	-
TIBC on NAFLD								
FinnGen	11	0.88 (0.68, 1.14)	0.84	0.86 (0.66, 1.13)	0.86 (0.66, 1.13)	0.88 (0.68, 1.14)	0.36	0.90
Leading liver centers	10	1.03 (0.89, 1.20)	0.40	1.05 (0.89, 1.23)	1.05 (0.90, 1.22)	1.03 (0.89, 1.19)	0.36	0.57
UK Biobank	13	0.90 (0.78, 1.04)	0.01	0.99 (0.86, 1.14)	1.03 (0.92, 1.16)	0.84 (0.70, 1.00)	0.80	0.21

**Meta-analysis**		0.95 (0.86, 1.05)	-	0.99 (0.90, 1.10)	1.02 (0.93, 1.11)	0.94 (0.85, 1.04)	-	-
NAFLD on ferritin								
Benyamin B et al.	7	1.05 (1.02, 1.08)	0.95	1.04 (1.01, 1.08)	1.04 (1.01, 1.08)	1.05 (1.02, 1.08)	0.27	0.95
Bell S et al.	6	1.01 (0.99, 1.02)	0.97	1.01 (0.99, 1.02)	1.01 (0.99, 1.02)	1.01 (0.99, 1.02)	0.52	0.97
**Meta-analysis**		1.01 (1.00, 1.02)	-	1.01 (1.00, 1.03)	1.01 (1.00, 1.03)	1.01 (1.00, 1.02)	-	-
NAFLD on iron								
Benyamin B et al.	7	1.01 (0.98, 1.05)	0.20	0.99 (0.95, 1.03)	0.98 (0.94, 1.02)	1.01 (0.97, 1.05)	0.13	0.21
Bell S et al.	9	1.01 (1.00, 1.02)	0.70	1.01 (1.00, 1.03)	1.01 (0.99, 1.03)	1.01 (1.00, 1.02)	0.63	0.66
**Meta-analysis**		1.01 (1.00, 1.02)	-	1.01 (0.99, 1.02)	1.01 (0.99, 1.02)	1.01 (1.00, 1.01)	-	-
NAFLD on TSAT								
Benyamin B et al.	4	1.08 (1.03, 1.12)	0.77	1.08 (1.03, 1.13)	1.08 (1.03, 1.14)	1.08 (1.03, 1.13)	0.99	0.85
Bell S et al.	7	1.02 (1.00, 1.04)	0.17	1.02 (1.01, 1.04)	1.04 (1.02, 1.06)	1.02 (1.01, 1.04)	0.09	0.22
**Meta-analysis**		1.03 (1.01, 1.05)	-	1.03 (1.01, 1.05)	1.04 (1.03, 1.06)	1.03 (1.01, 1.05)	-	-
NAFLD on TIBC								
Bell S et al.	5	1.03 (1.01, 1.05)	0.39	1.04 (1.01,1.07)	1.04 (1.01, 1.07)	1.03 (1.01, 1.05)	0.15	0.50

IVW: Inverse variance weighted; MR RAPS: MR Robust Adjusted Profile Score; MR PRESSO: MR Pleiotropy Residual Sum and Outlier; Q test: Cochran's Q statistics; OR: Odds Ratio; CI: Confidence Interval; NAFLD: Non-alcoholic fatty liver disease; TSAT: Transferrin saturation; TIBC: Total iron-binding capacity.

Table S2 Heterogeneity and test for overall effect of the IVW meta-analysis.

Meta-analysis (Fixed-effect)	Heterogeneity		Test for overall effect	
	I^2	*P*-value	Z	*P*-value
Ferritin on NAFLD	0%	0.650	2.62	0.009
Iron on NAFLD	0%	0.421	2.50	0.012
TSAT on NAFLD	0%	0.372	2.34	0.019
TIBC on NAFLD	0%	0.367	-1.07	0.284
NAFLD on ferritin	82%	0.018	2.71	0.007
NAFLD on iron	0%	>0.999	2.05	0.041
NAFLD on TSAT	83%	0.015	3.32	0.001

IVW: Inverse variance weighted; NAFLD: Non-alcoholic fatty liver disease;
TSAT: Transferrin saturation; TIBC: Total iron-binding capacity.
